# Implications of HPV infectivity in early diagnosis and treatment of advanced/recurrent malignancies

**DOI:** 10.1038/s44276-024-00036-y

**Published:** 2024-01-29

**Authors:** Takuma Hayashi, Ikuo Konishi

**Affiliations:** 1 Cancer Medicine, National Hospital Organization Kyoto Medical Center, Kyoto, 612-8555 Japan; 2 Medical R&D Promotion Project, The Japan Agency for Medical Research and Development (AMED), Tokyo, Japan; 3 Kyoto University Graduate School of Medicine, Kyoto, Japan

## Abstract

Human papillomavirus (HPV) genotype infection causes cervical cancer, pharyngeal cancer, etc. The amount of circulating HPV DNA in the blood helps in detecting advanced/recurrent cancer earlier than with tumour marker elevation. Cancer immunotherapy is more effective for HPV infection-positive than for HPV infection-negative cancer.

Human papillomavirus (HPV) causes sexually transmitted diseases and skin disorders. Its main route of transmission is sexual intercourse (penovaginal sex, oral sex, and anal sex) [1]. Latent HPV infection develops when the virus enters basal cells from a small genital wound. Sexually transmitted HPV infection is associated with development of benign condyloma acuminata, as well as pharyngeal, cervical (including vaginal), anal, and penile cancers [1]. In most people with HPV infection, the virus is eliminated by the immune system. However, in rare cases, HPV infections have been observed to persist for long periods. Clinical and basic medical studies to date have revealed that normal cells gradually become cancerous due to persistent HPV infection.

HPV vaccines prevent persistent infection by certain HPV types. Three HPV vaccines are prescribed in clinical practice: bivalent “Cervarix” (GlaxoSmithKline K.K., London, UK), tetravalent “Gardasil” (Merck KGaA, Darmstadt, Germany), and nonavalent “Sylgard 9” (Merck KGaA,). Clinical trials conducted in various countries have revealed that development of pharyngeal, cervical (including vaginal), anal, and penile cancers, as well as the benign disease condyloma acuminata, can be prevented by inoculation with a tetravalent or nonavalent HPV vaccine [2]. Globally, infections by HPV16 and HPV18 account for approximately 70% of all cervical cancers.

The treatment methods for a total of 2891 patients (OncoGuide^TM^ NCC oncopanel test: 763 patients and FoundationOne® CDx test: 2128 patients) were examined in cancer genomic medicine at Japanese national universities from December 2019 to January 2023 (Supplementary Material). A total of 36, 104, 22, and 10 patients with advanced pharyngeal, cervical, anal, and penile cancers, respectively, were studied (Supplementary Material). A cancer genomic test revealed that HPV16 or HPV18 infection was detected in 11 (11/36, 30.6%), 37 (37/104, 35.6%), 8 (8/22, 36.4%), and 3 (3/10, 30.0%) patients with advanced pharyngeal, cervical, anal, and penile cancers, respectively. Previous clinical trials have investigated the efficacy of nivolumab in 11 and 25 patients with HPV-positive and HPV-negative pharyngeal cancer, respectively. The results revealed that nivolumab was more effective at treating the patients with HPV-positive pharyngeal cancer than it was for the patients with HPV-negative pharyngeal cancer [3, 4]. The efficacy of immune checkpoint inhibitors, such as nivolumab, is significantly greater in malignant tumours with a high tumour mutation burden (TMB) or high microsatellite instability (MSI). Therefore, whether HPV infection increases the TMB and/or MSI of malignant cells is being investigated. Based on these clinical findings, clinical application of novel antitumour immunotherapies, which involve infecting malignant tumours with pseudoviruses and increasing the anti-immunogenicity against malignant tumours, is being considered [5, 6].

Cancer genomic testing is performed during medical care in Japan for patients with advanced cancer for whom standard or recommended treatments for various cancer types are not applicable. Therefore, the infection rate by HPV16 or HPV18 in advanced cancers is different from the incidence of cancer due to HPV infection in the Japanese population. However, the incidence of cancer caused by HPV16 or HPV18 infection (i.e., pharyngeal cancer, cervical cancer, or anal cancer) is expected to considerably decrease if HPV vaccination becomes widespread among the Japanese population. Clinical studies have reported that the number of cases of HPV-related pharyngeal cancer in males is approximately three times the number of cases of that in females. Therefore, HPV vaccination for males must also be widely available [7].

Recent clinical research has revealed that recurrence of various malignant tumours can be confirmed earlier by detecting circulating DNA in the blood derived from malignant tumour cells than by identifying increased tumour marker values [8]. Therefore, recurrence of HPV-related infectious malignant tumours can be confirmed earlier through detection of tumour-derived circulating HPV genes in the blood than through detection of increasing tumour marker levels [9] (Fig. 1). In particular, tumour-derived circulating HPV DNA is a useful marker for detecting malignant tumour recurrence versus tumour marker elevation.

**Fig. 1:**
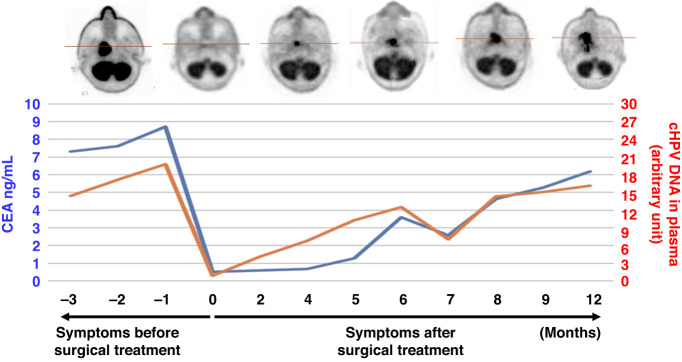
Early diagnosis of advanced/recurrent malignant tumours by detection of circulating HPV DNA rather than by detection of elevated tumour marker values.

Human oncogenes, such as Kirsten murine sarcoma virus, are well known as oncogenes derived from viral genes. Various malignant tumours caused by viral infection have been recognised, as previously mentioned. Vaccination is effective at preventing viral infection. However, unlike in bacteria, mutations in viral genes easily change the structure of viral backbone proteins and structural proteins. Therefore, the reduced preventive effect of vaccines against viral infections has become a problem. Additionally, producing vaccines that induce production of antiviral antibodies recognising the structural proteins of mutant virus variants is needed.

## Conclusion

Unlike treatment of bacterial infections by prescribing antibiotics, prevention of viral infections through vaccination and treatment of patients with drugs is challenging due to repeated mutations in the genes of viruses. However, the effectiveness of cancer immunotherapy has increased because malignant tumours are infected with HPV. Additionally, detecting circulating HPV DNA is more effective than identifying elevated tumour marker values for early diagnosis of advanced/recurrent malignant tumours. Furthermore, advances in medical technology have enabled early diagnosis and treatment of advanced/recurrent malignant tumours and are expected to improve medical technology innovations for cancer treatment.

## Supplementary information

Supplementary files
