# Supplementary material for: Implications of HPV infectivity in early diagnosis and treatment of advanced/recurrent malignancies
Source: BJC Rep. 2024 Jan 29;2:7. doi: 10.1038/s44276-024-00036-y (PMC11524121; doi:10.1038/s44276-024-00036-y)
Supplement: Supplementary file 1 — Supplementary files [file 44276_2024_36_MOESM1_ESM.pdf]

Supplementary files

Implications of HPV infectivity in early diagnosis and treatment of advanced/recurrent malignancies

Takuma Hayashi, Ikuo Konishi.

Supplementary material

1. Materials and Methods

1.1. Clinical Research

The abovementioned anti-Programmed death receptor-1 (PD-1) trials were included in the analysis with group arm from A multicenter retrospective observational study of patients who underwent an expert panel trial as chemotherapy control. The major assessed outcomes were adverse event (AE) and objective response rate (ORR). AE and ORR data were pooled up per regimen and described in percentage. The comparative incidences of AE between different regimens were evaluated using Fisher's exact test. Given that ORR of PD-1 blockade may differ according to treatment lines (firstlinevs. >1st line), we also evaluated the anti-PD-1 drugs per treatment setting and considered them as independent comparative groups when data was available. Given the evidence that infection with human papilloma virus (HPV), i.e., HPV 16, HPV 18, etc., tended to be associated with favorable responses to PD-1 blockade in NPC, we further evaluated the pooled ORR of anti-PD-1 therapies stratified by HPV positivity. Statistical analyses were performed using R version 3.5.1 (http://www.rproject.org). A two-tailed $P < 0.05$ was considered statistically significant.

1.2. Genome analysis using cancer genome gene panel

A total of 108 NPC patients were screened from 18 hospitals beginning on 10 December 2019. Eligible participants met these criteria: (i) confirmed NPC; (ii) received 1 dose of anti-PD-1 treatment; (iii) available medical record and willingness for follow-up. Clinical and demographical data were collected at the enrollment. The last date of follow-up was 10 October 2022.

OncoGuide™ NCC oncopanel*; Gene mutation analysis set for cancer genome profiling test (Sysmex Corporation Kobe, Hyogo, Japan), Foundation One CDx**; Foundation One CDx's cancer genome test (Foundation Medicine, Inc., Cambridge MA, USA)

1.3. Statistical Analysis

All data are expressed as mean ± SEM. Normality was verified using the Shapiro-Wilk test. Statistical analyses were performed using an unpaired two-tailed *t* test or Mann-Whitney *U* test for comparison of two groups. For multiple comparisons, one-way analysis of variance (ANOVA) with a Tukey post hoc test or a Kruskal-Wallis analysis with a post hoc Steel-Dwass or Steel test was used. A $p < 0.05$ was considered statistically significant. All statistical analyses were performed using JMP software (SAS Institute, Cary, NC, USA).

1.4. Ethical approval and consent to participate.

This study was reviewed and approved by the Central Ethics Review Board of the National Hospital Organization Headquarters in Japan (Tokyo, Japan) and Shinshu University (Nagano, Japan). The exact date when the ethical approval was obtained was August 17, 2019. The code number of the ethical approval was NHO H31-02. The authors attended educational lectures on medical ethics in 2020 and 2021, which were supervised by the Japanese government. The completion numbers for the authors are AP0000151756, AP0000151757, AP0000151769, and AP000351128. Consent to participate was required as this research was a clinical research. Subjects signed the informed consent when they were briefed on the clinical study and agreed with contents of clinical research. The authors attended a seminar on the ethics of experimental research using small animals on July 02, 2020 and July 20, 2021. They became familiar with the importance and ethics of animal experiments (National Hospital Organization Kyoto Medical Center and Shinshu University School of Medicine). The code number of the ethical approval for experiments with small animal was KMC R02-0702.

1.5. Author Contributions.

T.H. performed most of the clinical work and coordinated the project. T.H. conducted the statistical analysis. T.H. conceptualized the study and wrote the manuscript. T.H., and I.K. carefully reviewed this manuscript and commented on the aspects of medical science. I.K. shared information on clinical medicine and oversaw the entirety of the study. All authors have read and agreed to the published version of the manuscript.
